# Cognition Without Neural Representation: Dynamics of a Complex System

**DOI:** 10.3389/fpsyg.2021.643276

**Published:** 2022-01-12

**Authors:** Inês Hipólito

**Affiliations:** Berlin School of Mind and Brain, Institut für Philosophie, Humboldt-Universität zu Berlin, Berlin, Germany

**Keywords:** neurocognitive activity, neural representation, dynamic causal modelling, dynamical systems theory, embodied, enactive cognitive science

## Abstract

This paper proposes an account of neurocognitive activity without leveraging the notion of neural representation. Neural representation is a concept that results from assuming that the properties of the models used in computational cognitive neuroscience (e.g., information, representation, etc.) must literally exist the system being modelled (e.g., the brain). Computational models are important tools to test a theory about how the collected data (e.g., behavioural or neuroimaging) has been generated. While the usefulness of computational models is unquestionable, it does not follow that neurocognitive activity should literally entail the properties construed in the model (e.g., information, representation). While this is an assumption present in computationalist accounts, it is not held across the board in neuroscience. In the last section, the paper offers a dynamical account of neurocognitive activity with Dynamical Causal Modelling (DCM) that combines dynamical systems theory (DST) mathematical formalisms with the theoretical contextualisation provided by Embodied and Enactive Cognitive Science (EECS).

*It will be all too easy for our somewhat artificial prosperity to collapse overnight when it is realized that the use of a few exciting words like information, entropy, redundancy, do not solve all our problems* (Shannon, 1956, p. 3).

## Introduction

In his seminal paper in 1995, Van Gelder (1995) asked, ‘what might cognition be, if not computation’. In that paper, he tells us that computationalism becomes so prominent due to the false assumption that there is no other option. The received view of cognition as machine-like computation dates back to the beginning of the cognitive revolution (for a review, see Miller, 2003). The brain-computer metaphor provided the conceptual toolkit that cognitivism^1^ greatly required to push back against the behaviourist explanation of mental events.

Many computer scientists and mathematicians contributed to the toolkit. One of them was von Neumann (1958, p. 3) defining computation as ‘to operate on … numbers according to a predetermined plan’. Despite his contribution to the toolkit, von Neumann (1958) always resisted the metaphor he helped create, granting the metaphor an ‘absolute implausibility’ on account of the distance between a biological system and a computer (Peters, 2018). However, as noted by Piccinini (2003, p. 329) because ‘Neumann did not clarify what he had in mind… many scientists adopted his language with the same lightheartedness’. Gradually the computer-brain metaphor metamorphoses into a literal sense by which neural activity *ought to be* machine-like computation. This reasoning becomes so pervasive in cognitive science that it becomes hard to see other options in the field. The orthodox attitude is well-captured in statement of Newell (1990, p. 56): ‘there do not seem to be any viable alternatives… to the computational theory of mind (CTM)’.

While neurobiological transduction could be seen as involving some *kind* of biological *computation*, following von Neumann (1958), given the distance between a biological system and a computer, biological activity should not be described or defined in the terms, we use to refer to how a computer computes. Manicka and Levin (2019) have shown that a minimal non-neural Bio-Electric Network model that utilises the general principles of bioelectricity (electrodiffusion and gating) can *compute*. This evidence motivates the reasoning that considering that (P1) neurobiological activity is neurochemistry of thermodynamic energy transfer, and if (P2) non-neural biological structures compute and (P3) neural structures compute, then (C) computation is neurochemistry of thermodynamic energy transfer. From this follows that if we must use the notion of *computation* to refer to neurobiological activity, it must be such that it applies to all scales of neurobiology, which means deflating the notion to the extent that it becomes nothing like a computer.

Linson et al. (2018), in their paper pushing against the traditional input/output understanding of computationalist cognition, claim that active inference, a predictive coding descending account of neurocognitive activity, ‘re-arranges the picture to dispense with the classical/computational model of input and output’ (p. 9). They conceive of neurobiological activation patterns through an analogy with a game of tennis. The act of hitting a ball with a racket would take much imagination to be conceived of in terms of an input to the ball. After all, what would be the output? The appropriate way of characterising this activity is, by using fundamental physics, ‘regarding the action of hitting the ball as a transfer of energy, from the arm to the racket to the ball’ (p. 9). Applying this example to how a system is influenced by its environment, for example, a cell in a tissue, a neuron in a cortical network, or a brain in an organism, then, thermodynamic energy transfer occurs between the local environment and the system, for example, between other neurons in a cortical network, or between cells, as computationally explained in Bio-Electric Network model of Manicka and Levin (2019).

Cognition, however, according to the orthodoxy of machine-like analogies, is conceived of as a product of a neuronal information-based process that unfolds to the end of computing intelligible representations (Fodor, 1983a; Millikan, 2017, 2020; Piccinini, 2018; Shea, 2018; Sprevak and Colombo, 2018; Smortchkova et al., 2020; Rupert, 2021). This view is so engrained in computational cognitive neuroscience that one would seem mad to question it. Some might say because computation ‘makes us blind’ (Brooks, 1991; see also Brooks et al., 2012). The lack of (an alternative) vision might be due to a phenomenon well-diagnosed by Egan (2020, p. 31), that ‘despite the fact that there is no widely accepted naturalistic foundation for representational content, computational theorists persist in employing representational language in articulating their models’. In cognitive neuroscience, it is not uncommon to hear that the concept of *representation* should be left for philosophers to deal with. But as Dennett and Dennett (1996, p. 21) well notes, ‘[t]here is no such thing as philosophy-free science; there is only science whose philosophical baggage is taken on board without examination’.

There are at least two main consequences of taking *representation* on board in neuroscience without examination. First, in the light of the brain-computer metaphor, neural and cognitive activity becomes seen as the transfer of symbolic information (recall definition of von Neumann, 1958). While metaphors can aid understanding to some extent by explaining by analogy some common feature between two objects, being a metaphor also means that not all features between the two objects will match, but that we can make a loose analogy, provided that we accept some levels of abstraction and fiction. The brain-computer metaphor serves the purpose of computers offering an abstract or fictional model of the brain. As an abstraction, it may aid some epistemic understanding of some part of the brain. But epistemology does not grant *per se* ontological properties. As did von Neumann (1958), one must resist the metaphor (Brette, 2019).

The second consequence of taking *representation* without examination is that it leaves out the possibility of aspects of cognitive and neural activity that are not machine-like computations. Adopting the machine language light-heartedly will at some point give the impression that either all that there is to mental life is computation (i.e., to operate on … numbers according to a predetermined plan); or even if there are some non-computational aspects to cognition, these are not relevant enough to cognitive neuroscience. This limitation has been widely acknowledged by non-representational approaches such as Embodied and Enactive Cognitive Science (EECS), contending that cognitive activity does not reduce to mental representations and rejecting the notion of neural representation altogether (Dreyfus, 2002; Maturana, 2002; Fuchs, 2017; Hutto and Myin, 2017; Gallagher, 2020). A related consequence has been crucially pointed out by Barrett (2016, p. 9). Placing the mark of the cognitive in computational processes results in that ‘a great injustice is done to both human and nonhuman animals: On the one hand, we fail to recognize the distinctive nature of nonhuman cognition, and on the other hand, we continue to promote a somewhat misleading view of human psychological capacities’.

## Models in Cognitive Neuroscience

Modelling science is the construction of computational models for the scientific understanding of a physical phenomenon that would otherwise not be possible to tackle, given its complexity and large degrees of freedom. In a way, a model is a substitute for empirical experimentation. Examples of phenomena tackled with models include any highly complex system such as the weather, the ocean, or the brain.

A scientific model is a physical and/or mathematical and/or conceptual representation of a system of ideas, events, or processes. Scientists seek to identify and understand patterns in our world by drawing on their scientific knowledge to offer explanations that enable the patterns to be predicted objectively. Computational modelling in cognitive science aims at explaining and understanding cognitive behaviour, such as emotion (Ong et al., 2019), decision making (Calder et al., 2018), (ethical) reasoning (McLaren, 2006; Demigha, 2020), planning, problem-solving, and moral behaviour (Allport, 1947) as well as psychopathologies such as depression, anxiety, eating disorders, or addiction.

Models in cognitive neuroscience are a link between what is known and what is unknown. More precisely, models aim to connect the data collected from behavioural or neuroimaging studies (what is known) with the activity that generated the data (what is unknown). In cognitive neuroscience, scientists use models to explain the activity that generated the collected data. More precisely, the model leverages, roughly speaking, a theory or a folk ontology constructed by the modeller that aims to explain how the data has been generated.


$$\begin{array}{r} Scientific\;Model\;\left(\mathrm{SM}\right)=data\;\left(D\right)+theory\;about\;how\;\\ the\;data\;was\;generated\;\left(T\right). \end{array}$$

Because a model is constructed to test a theory about how the data was generated, many in philosophy of science claim that a model is a representation. It is argued that a model, being the product of a scientific endeavour, is a scientific representation (Suárez, 2010; Poznic, 2018; Beni, 2019; Frigg and Nguyen, 2020). While there is much debate about representation in science, the problem comes roughly as discussions about the adequacy of an account of scientific representation (Camardi, 2020; San Pedro, 2021).

Some scientists and philosophers are compelled to add yet another layer. They suppose a metaphor between the work of a scientist constructing models/representations and neuronal activity (Poldrack, 2020). This means that the brain at work also theorises, constructs, and tests a model, thereby delivering representations. Neural connectivity distributed in computations, just like the work of a scientist, delivers representations with important causal roles. Neural activity is explained as if it were literally a computation on information-based mechanisms. From this perspective, it is not only the scientist who constructs representations about a target system, but the target system also constructs representations. To do so, neurons are supposed to encode information and compute information to deliver a representation that is then meaningful for another neuron in the network chain of information processing. Examples of approaches in computational neuroscience taking this view include neural networks as topological mappings of the connectivity and activity in the nervous system. As a map of the connectivity and activity, a model of the neural network of the nervous system is said to represent it (Sporns, 2007; Hagmann et al., 2008; Bullmore and Sporns, 2009; Rosenthal et al., 2018; Goulas et al., 2021). The process is analogous to scientists constructing and delivering representations that are meaningful to the scientific community.

While, as we will see, this metaphor is at the basis of cognitive neuroscience (i.e., neural networks, deep and machine learning, and cognitivism) and philosophy of mind (CTM, connectionism), the metaphor between scientific computational models and neurons that compute representations is not held across the board is computational neuroscience (Freeman and Skarda, 1990; Brette, 2019; Friston, 2019; Jafarian et al., 2021).

The following section explains the two primary techniques of modelling brain activity as neural connectivity. It is shown that because these techniques hold different assumptions about whether or not there are representations in neural activity, they tackle completely different questions (*how* vs. *why* processes), subject matters (topology vs. causes), and elucidation goals (description vs. explanation), respectively. Finally, it is stressed that these are two ways in which neuronal connectivity can be made intelligible to us. While models of brain connectivity correspond to a link between known data (the data collected) and simulation of the unknown data (the activity that generated the data), neuronal and cognitive activity as the target that needs to be explained. So, models of brain data and cognitive processes are irreducible to one another. This means that while there could be representational properties in the scientific model, it does not follow that there are symbolic representational properties in neuronal activity.

## Scientific Models in Neuroimaging

In neuroscience, models are valuable tools for the understanding of brain activity in terms of connectivity. Functional neuroimaging aims to depict the function of the central nervous system with the support of a network of models of cortical activity. As an evolving field, the methods of functional neuroimage today reveal active distributed networks in a way that no other method has done before.^2^

Some of the core difficulties in functional neuroimaging remain. These are due mainly to the vast amount of data collected from the brain scan. Models are helpful to attempt to make sense of what activity generated the data. This understanding contributes to developing a framework of the physiological relations between blood flow, neuron discharge, and energy metabolism and how this physiological activity relates to how psychological activity unfolds. This is, in essence, the problem of how to link neuronal and cognitive activity.

The critical issue is establishing relevant activity and how to pattern the activity in an intelligible way. Models in cognitive neuroscience aim to link what is known and what is unknown. More precisely, models aim to connect the data collected from neuroimaging studies (what is known) with the activity that generated the data (what is unknown). Put otherwise, to attain the functions and processes from the vast amount of brain data (e.g., whole brain); we require models of data for the neuronal influences. A model of data is an idealised version of the raw data, we gain from functional neuroimaging. The main goal of a data model is to confirm a theory, considering that the model of the collected data is what a theory is compared against, not the messy, complex, raw data. In other terms, the model aims to confirm a theory that motivated the model. In short, a model corresponds to a theory, that is to say, a theory about the connections observed.

Models of data in neuroscience can be conceptually organised into two broad categories: those assuming and rejecting neural representations. The former I shall call *structural information models* (SIM) and the latter, *dynamical models* (DM). SIM are right at the core of brain mapping, aiming to develop a model that displays the rules by which information is either propagated or constrained within structures in the brain. The distributed information in causal structures is typically inspired by connectionism and, more recently, is the view endorsed by neural networks and deep learning using Structural Causal Modelling (SCM) to map connectivity in terms of presence or absence of ‘information’ in a neuronal structure at a given time (Bielczyk et al., 2019; Borsboom et al., 2019; Straathof et al., 2019). As such, SCM aims to offer easy-to-use maps depicting the topology of causal connections amongst the neuronal parts exchanging ‘information. This is why, for these models, acyclic graphs are sufficient and easy to use. Instead of the direction of the causation, these graphs offer another indication. The width of the edges indicates how likely it is for a region to connect to another, where a thicker edge means increased likelihood. Dependency graphs or Bayes nets depict causal links through directed edges (Lauritzen, 1996; Pearl, 1998; Spirtes et al., 1998).

**Figure 1:**
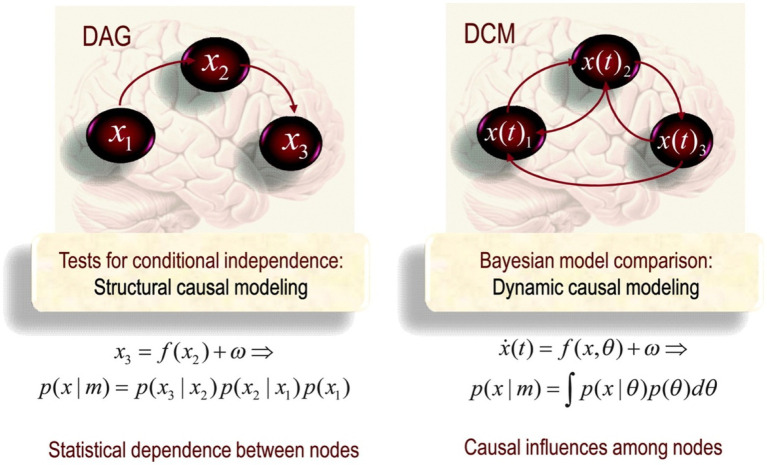
Schematic highlighting the distinction between Direct acyclic graph (DAG) (on the left) and Dynamical Causal Modelling (DCM; on the right) in the form of a directed cyclic model (Friston, 2011, p. 25).

Structural Causal Modelling is conventionally used to pick out conditional independence, underlying functional connectivity, between nodes in a network. For example, in Figure 1, X3 is statistically independent of X1 given X2. This means it is possible to understand the properties and dynamics of X3 independently of X1 if we know X2. SCM has a particular topological ordering without any directed cycles between nodes. One important point to note is that causation in SCM fails for phenomena involving distant correlations as it breaks with the idea of large-scale causal connectivity between multiple nodes in a network. By being motivated to describe local pairwise connections, X1 and X2, SCM is not concerned with large-scale functional architectures. On the contrary, the fundamental question driving functional analysis is how likely it is for areas to connect in a certain way, given certain (physiological) conditions.

By determining sequential correlations, it is possible to determine which nodes are functionally segregated. Notably, these elements are arranged linearly or sequentially in indirect graphs, where two elements stand in correlation with each other. As essentially a statistical measure, functional connectivity does not rest upon any model of statistical dependencies among observed responses. In functional analyses, there is no inference about what motivates the coupling between X1 and X2. Instead, the inference is about whether X1 and X2 are correlated, i.e., disambiguating between statistical dependency and the null model (hypothesis) of no dependency, stipulated in terms of probability distributions over observed multivariate responses (This goal is achieved by analysing brain networks *via* acyclic graph metrics describing functional connectivity). SCM, under SIM, provides a rigorous set of tools to quantitatively describe topological properties of complex systems such as the brain (Rubinov and Sporns, 2011; Rathkopf, 2018). The methodological tools employed to allow for the extraction of different kinds of correlational information from the topology observed at different spatial or time scales; which thereby allows, following Sporns (2011, p. 29), ‘for a more comprehensive analytic strategy for investigating the neural substrates of cognition’. Under *information modelling* or *theory*, cognition lies in highlighting ‘the subsets of network nodes that are more densely interconnected among themselves than they are with other nodes in the network’ (p. 32). SIM implements SCM methodologies to capture the conditional independencies between the elements of a network.

In conclusion, SCM rests upon a theory of dependencies, such as correlations or coherence between remote neurophysiological events. Even in its dynamical form, its aim is not to provide a causal explanation of the sort provided by (model-based) effective connectivity but to display a causal topology. Because, theoretically, these models assume neuronal connectivity as information and its display, the theory itself does not call for an explanation of the reasons why connections occur in the ways they do. SCM is, in conclusion, a modelling set of techniques typically employed in the topological mapping of connectivity.

Dynamical models take a different approach. They take neuronal connectivity as interaction instead, speaking to effective connectivity.^3^ In DM, neuronal connectivity is cast instead as the presence or absence of interaction between parts of a system. The aim is to develop a model and test the theory that elucidates upon the observed data and assists in making sense of it as patterns according to a principled explanation. As opposed to the topological description of the information category of modelling, the interaction category of models is interested in testing the theory that contains a hypothesis that explains why a specific neuronal activity occurs or what motivates it.

Dynamical causal models (DCM) are used to investigate the context-sensitive, direct coupling of neuronal connections, targeting the influence explicitly that one neural system exerts over another. Neuronal activity is taken as interactions that unfold over time in changing, highly contextual environments that present features of chaos and unpredictability. Causes of neural coupling are precisely the subject matter of effective connectivity (Zarghami and Friston, 2020). At the root of this enquiry are questions about what is relevant to determine the causes underlying neural connections. Effective connectivity emphasises the observed dependencies between regions concerning what motivates the coupling or directed influence between them. To do so, it rests explicitly on a model of that influence – called a generative model. Notably, each model corresponds to an alternative hypothesis about how the correlation, say between A and B, at a synaptic or populational level, was caused.^4^ These models are optimised to fit measured data and then compared based upon their relative (Bayesian) model evidence.

From this perspective, DCM attempts to answer the specific methodological difficulty in neuroimaging regarding how to link neuronal and cognitive activities under the same patterned explanation by explicitly clarifying the influence that one neural system exerts over another, i.e., the direction of the causation between systems. Dynamic models of effective connectivity are thus an inference or theory about the most likely cause of a correlation of dynamics. More precisely, they are a generative model of how statistical dependencies and independencies are generated. The inference about the cause of the correlation between neural systems is made by introducing latent (neuronal) states. That is, by introducing a state of context-dependent coupling into a model of interactions. This means that these models feature a state-dependent causal architecture, in which condition-specific changes in connectivity are usually of primary interest. On this account, models of effective connectivity are dynamic – they do not suppose that there is an invariant structure whose plastic connections can be topologically mapped. But there is a dynamic structure that is pressured by activity, where the explanatory traction can be gained. Specifically, by two crucial features: dynamics (activity-dependent) and a model of directed interactions or coupling. Effective connective is thus model-based, i.e., can be reduced to model comparison. That is to say, the comparison between models to infer its explanatory accuracy to observed functional connectivity. In this sense, effective connectivity analysis recapitulates the scientific process (Zarghami and Friston, 2020). Accordingly, research on effective connectivity requires tools to explain what motivates activity-dependent effects on synaptic connections. For that reason, DCM seems suitable as it applies nonlinear dynamics to model the activity in the nervous system, from single neural cells to cognitive interaction with the environment.

A dynamical model thus needs to encompass all possible states, expressed by a phase state, of neurons in a large-scale neuronal and cognitive simulation. DCM is a technical means for simulating this. Under the assumption of different causal interactions, it applies model comparison and selection to develop models to investigate the coupled influences among the other parts of the nervous system and what drives the re-organisation of neuronal activity. Considering that neuronal connectivity is spatiotemporally situated, reciprocally in a spatiotemporal dynamic that comprises and contributes to the system’s history, direct cyclic graphs are a more suited tool. Cyclic graphs are formally equivalent to those used in SCM; however, in DCM, they are used as explicit generative models of observed responses. The arrow in effective connectivity denotes the temporal asymmetry denoting causality (see Figure 1). The task of neuroimaging is thus to capture the neuronal activity in terms of dynamical states and draw patterns of temporal and spatial activity. Because of the dynamical understanding of neuronal activity, DCM is consistent with dynamical and complex systems theory (DCST) and EECS.

Thus far, the paper contended that theories in brain data analysis are conceived under two main categories: those taking brain activity as ‘information’ and those casting it as dynamical states. Either conception is purely theoretical. It is a theory about interpreting brain data: either as ‘information’ or states. The former is helpful in many modelling scientists because the goal is to unfold a topological map describing the likelihood of connectivity. The latter is a theoretical understanding of neuronal activity in terms of why connections occur the way they do.

In conclusion, while the two main modelling techniques are motivated by different theoretical assumptions, they set off completely different paradigms tackling different questions (*how* vs. *why* processes occur), subject matters (topology vs. causes), and elucidation goals (description vs. explanation), respectively. Both models, however, aim to solve a common problem: to explain how the data has been generated. They do so by linking what is known (neuroimaging data) with a theory about what is known (how the brain has generated the data). These models are thus a result of data plus a theory about the data. As stated in section Scientific Models in Neuroimaging:


$$\begin{array}{c} Scientific\;Model\;\left(\mathrm{SM}\right)=data\;\left(D\right)+theory\;about\;\\ how\;the\;data\;was\;generated\;\left(T\right). \end{array}$$

Neurocognitive activity is not a result of data plus a theory about data, but the phenomenon itself that the scientists aim to explain. Brain activity is not a result of data and a theory of data. Models of brain data and brain activity are thereby not reducible to one another. In short, models of brain data are not analogous to brain activity; they should not thereby be conflated in any form or kind of metaphor: for this would be fallacious reasoning. This is fallacious reasoning because even if there are representational structures in the models of the brain because models of the brain and brain activity are irreducible to one another, it does not follow that there are representational structures in the brain.

The following section shows how, even though brain models and brain activity are two different things, non-reducible to one another, the history of philosophy of mind writes itself on the assumption that they are analogous. More precisely, assuming that properties of models of the brain, i.e., information, semantics, syntax, functions, models, representations, etc., can and should be applied to explain the mind.

## Philosophy of Mind and Its Computational Dashes of Realism

The previous section shows two major ways computational neuroscience develops models for understanding neural activity. The history of philosophy of mind goes hand in hand with computational models, most times applying the conceptual machinery of the time to the mind. Examples of this, we will see below, include the computational theory of mind, connectionism, and, more recently, the Bayesian brain.

The rise of Turing computing stimulated Fodor (1983b) into applying its computational concepts and technicalities to the explanation of the mind. A Turing machine (Turing, 1936) is a modelling computation of input/output instructions that manipulate symbols according to a set of predefined rules. In this kind of modelling, given any algorithm, it is possible to generate the algorithm’s logic. Technically, a Turing machine comprises operations in discrete cells or modules. The machine’s ‘head’, positioned over a cell, ‘reads’ the symbol in the cell. The ‘head’ then makes sense of the symbol by referring to the action table of rules, and either proceeds to a subsequent instruction or halts the computation.

Fodor applies Turing’s constructs to the explanation of the mind.^5^ The mind, Fodor contends, comprises computations in a realist sense, with a central system and satellite, modular systems (Fodor, 1983b). The CTM, scaffolded on a realist account of Turing machines, holds that the mind is a computational system that is realised (i.e., physically implemented) by neural activity in the brain (Horst, 2003; Rescorla, 2015; Gallistel, 2017; Rabkina et al., 2017). The CTM is not a metaphor between a computer and the mind. It is the view that the mind is, literally, a computer system processing information. More precisely, ‘it states that a computer simulation of the mind is sufficient for the actual presence of a mind and that a mind truly can be simulated computationally’ (Rescorla, 2020). Like a Turing machine, the mind is a symbolic operator and mental representations are symbolic representations, just like language meanings. On a modular architecture, symbolic representations are computed in modules; their processing involves automaticity, mandatory operations, fast processings, shallow outputs, and fixed architectures.

Building upon this, analytic philosophy of mind comes to a discussion about the representation in this computational device. How should representation be understood? Classic representationalists believe that mental representations are symbolic structures with semantically evaluable constituents and that mental processes are rule-governed manipulations that are sensitive to their constituent structure (Turing, 1950; Newell and Simon, 1972; Marr and Vaina, 1982; Fodor, 1983b; Fodor and Pylyshyn, 1988). Teleosemantics, accordingly, tells us mental representations should be understood in terms of biological functions allowing for some role of the environment to shape symbolic structures (Neander, 2017; Garson and Papineau, 2019; Shea, 2020). Millikan (2020, p. 1) tells us that, ‘[c]orrectly understood, teleosemantics is the claim that ‘representation’ is a function term. Things are called ‘representations’ if they have a certain kind of function or telos and perform it in a certain kind of way’,^6^ where the telos is consistent with Turing’s action table of rules.

The rise of distributed computing brings up a revolution in the understanding of the mind. Distributed computing is a system whose components are located in different networked computers, which communicate and coordinate their actions by passing messages to one another. Distributed systems interact with each other to achieve a common goal in a distributed program. Distributed computing is motivated to solve a computer problem. Here, the problem is divided into many tasks, each of which is solved by one or more computers, which communicate *via* message passing.

Cognitive scientists applied this form of computing to explain the nervous system (and linked cognitive activity) in a model called Parallel Distributed Processing (PDP) (McClelland and Rumelhart, 1986).^7^ These were the early days of neural networks (Cowan, 1990). PDP inaugurated new ways of simulating and getting insights into how the nervous systems work, known as *connectionism*. With this new programming tool, it is then possible to model the nervous system as an electrical network of neurons with good results for sensory perception and motor activity simulation. Connectionists, taking a literal sense of PDP modelling, hold the model in a realist way commit to the view that mental representations are realised by patterns of activation in a network of simple processors (or nodes), just like in the models computation scientists develop. The main difference between PDP and Turing computing is how representation should be understood. Representations in nodes are not necessarily semantically evaluable, nor do their patterns of activation have semantically evaluable content. Nevertheless, some ‘localist’ versions somewhat aligned with connectionism, like that held by Ballard (1986), conceive representations in individual nodes as having semantic properties.^8^ PDP is revolutionary because its advent allowed for developing novel ways of developing tools to simulate the brain and make sense of data collected from neuroimaging.

This gives rise to a new computational model: predictive coding. Predictive coding is the theory in computational neuroscience that aims to explain the brain’s activity as constantly generating a model of the environment. The model is used to generate predictions of sensory input by comparing it to actual sensory input (Shipp et al., 2013; Spratling, 2017; Friston, 2018; Smith et al., 2021). Bayesian formalism is used as a rule to update the model as new information becomes available. As we will see, predictive coding has set off many ships in cognitive science and philosophy of mind, which include the *conservative* prediction error minimisation (PEM; Hohwy, 2013, 2018, 2020; Gładziejewski, 2019, 2021; Kiefer and Hohwy, 2019; Litwin and Miłkowski, 2020; Seth and Hohwy, 2021), the *radical* predictive processing (PP; Orlandi, 2014; Clark, 2015), and active inference (Friston, 2013). The *conservative* PEM and the *radical* PP, while agreeing on the analogy between the brain and a predictive machine, thereby applying conceptual machinery as an ontological property of brain activity, differ in how they understand the isolation or encapsulation of this machine brain. For PEM, the brain is a machine of PEM that is sufficiently equipped to construct and update models of the world, where ‘the mind can be understood in internalist, solipsistic terms, throwing away the body, the world, and other people’ (Howhy, 2016, p. 7). While Hohwy (2016) encapsulates mental life in the skull, Clark (2015) deflates the notion of representation. Clark (2015, p. 4) takes it ‘as minimal as possible, neither encoding nor processing information in costly ways’. The reason for this is that there is yet another inference/representational model: top-down motor prediction. In the end, both neuronal and cognitive activity comes down to inference mechanisms and models (Kirchhoff et al., 2018; Miller and Clark, 2018; Kiverstein et al., 2019; Wilkinson et al., 2019), a framework that has received its criticisms (Gallagher et al., in press; Hutto and Hipólito, in press; Hutto, 2018; Williams, 2020).

Finally, active inference, also a descendent from predictive coding and Bayesian inference, is a modelling tool that explains a living system’s activity as minimisation of uncertainty, entropy, or surprisal (Parr et al., 2022). It is a corollary of the Free Energy principle stating that the evolution of systems, i.e., how a system interacts with the environment to maintain its integrity, can be understood as the internal states free energy gradient descent by using variational Bayesian methods (Da Costa et al., 2020). According to active inference:

Internal states (and their blanket) *will appear to engage in active Bayesian inference*. In other words, they *will appear to model* – and act on – their world to preserve their functional and structural integrity, leading to homoeostasis and a simple form of autopoiesis (Friston, 2013, p. 1).

The internal states of a system of interest and the external states will indirectly influence one another *via* a further set of states: active and sensory states (called blanket states). These influences can be explained by supposing that internal states of a system engage in an active inference activity: that of predicting the external state. A probabilistic model predicts how external states influence the Markov blanket that is implicit in the dynamics of internal states: a *generative model*. While active inference is a formalism that can be used to construct scientific models in terms of variational free energy, i.e., as an information-theoretical function that can be applied to solve for optimisation of model evidence and comparison analysis, it does not follow that everything in every scale of life that can be modelled by active inference, literally engages in active inference. Supposing this requires a philosophical argument.

What all these theories in philosophy of mind, from the computational theory of mind, connectionism, PEM, PP, and active inference, share is the tendency to hold the analogy between the mind and a machine-like computer and its activity with computational models, be it symbolic, distributed processing, or statistics predictive processing. This means two things: (1) that the properties of the computational model construed are expected to exist in the target phenomenon, and (2) that claims made about the computational model should also hold for the target system. A difficulty of endorsing (1) and (2) is that, for consistency, one should expect this to be the case when the target is not brain and/or cognitive activity. After all, classic, connectionist, and Bayesian computational models are widely applied for understanding and predicting the behaviour of other complex phenomena (e.g., weather, societies, etc.).

Prediction error minimisation, PP, and active inference give rise in philosophy of mind of a new trend resulting, if taking a realist standpoint, mainly from the literal application of predictive coding. As I have argued elsewhere, the literal sense is not necessary: for rejecting the analogy between the mind and brain as a computer and its processes as computational models, computational models become an instrumental tool that, once applied to a behavioural activity, allows the scientist to draw interpretations and explanations, but the system under study does not have the properties of the model (van Es and Hipólito, 2020).

The history of analytic philosophy of mind is written with many examples of applying concepts of modelling neuronal activity to the explanation of mind and cognition (Turing, PDP, Bayesian modelling are examples). Over the years, philosophy of mind has adopted much of the conceptual machinery and insights of the best computational modelling of the time, for example, Turing machines, PDP, or Bayesian modelling, giving rise to the computational theory of mind (Fodor, 1983b), connectionism (Bechtel and Abrahamsen, 1991), and the Bayesian brain (Rescorla, 2019) and predictive accounts of cognition (Hohwy, 2013; Clark, 2015), respectively.

The project of providing a theory about the mind by borrowing conceptual machinery from computational neuroscience will be doomed if computational neuroscience returns this project back to philosophy. Egan (2020, p. 31) makes a similar point: ‘the majority of philosophers of mind who look to the cognitive sciences, and in particular, to computational neuroscience, to provide a naturalistic explanation of our representational capacities. Their hopes would be dashed if cognitive science just kicks the project of naturalising the mind back to philosophy’. But if turning to computer science for answers about the mind must be the case, then one should also pay attention to important remarks made by computer scientists. Shannon (1948), the visionary of information theory, explicitly points out that his theory concept does not apply to human brains and cognition. In his ‘bandwagon’ paper (Shannon, 1956), he expressed his discomfort, here quoted in full:

Although this wave of popularity is certainly pleasant and exciting for those of us working in the field, it carries at the same time an element of danger. While, we feel that information theory is indeed a valuable tool in providing fundamental insights into the nature of communication problems and will continue to grow in importance, it is certainly no panacea for the engineer or, a fortiori, for anyone else. Seldom do more than a few of nature’s secrets give way at one time. It will be all too easy for our somewhat artificial prosperity to collapse overnight when it is realized that the use of a few exciting words like *information*, *entropy*, and *redundancy*, do not solve all our problems. What can be done to inject a note of moderation in this situation? In the first place, workers in other fields should realize that the basic results of the subject are aimed in a very specific direction, a direction that is not necessarily relevant to such fields as psychology, economics, and other social sciences… if, for example, the human being acts in some situations like an ideal decoder, this is an experimental and not a mathematical fact, and as such must be tested under a wide variety of experimental situations (p. 3, highlights in the original).

What Shannon is concerned about is the danger of confusing the map with the territory, and precisely moderate the claims, especially those about psychology and biology, that can be made solely on the account of the maths. Cronbach (1955) shares Shannon’s concerns arguing against the indiscriminate application of information theory to topics in psychology (for a review, see Sayood, 2018). While Shannon shows lucid reasoning, the assumption of information (and computations over it) in the brain is pervasive (Reinagel, 2000; Stone, 2018; Nizami, 2019; Luppi and Stamatakis, 2021), the debates revolving around what *kind of information is brain information* (Rathkopf, 2020): semantic, Shannon, or something in between? As noted by Shannon, applying information theory to the brain would be a simplistic description that is nevertheless widespread, preventing the progress in a theory of the mind and cognitive and brain science. A new theoretical breakthrough is needed, one that is not at the mercy of the technological developments in computer science.

## What Might Cognition be, If not Neural Representation?

Embodied and Enactive Cognitive Science (EECS), as a theoretical framework and Dynamical and Complex Systems Theory (DCST), as a formalism, is widely known for rejecting the metaphor between cognition and computers. Both accounts, EECS and DCST, categorically reject the talk of the mind as processing a device:

*We deliberately eschew the machine vocabulary of processing devices, programs, storage units, schemata, modules, or wiring diagrams*. We substitute, instead, a vocabulary suited to fluid, organic systems, with certain thermodynamic properties (Thelen and Smith, 1996, p. Xix, emphasis added).

These two accounts, EECS and DCST, although quite aligned in their rejection of the mind as a computer analogy, do not always work together. This section aims to bring together these two frameworks and show how explicit cooperation fulfils a cohesive programme in cognitive science.

Embodied and enactive cognitive science rejects the existence of representations or any form of model-like theorising at any level that is not the level of a fully enculturated agent. More precisely, an agent that has been enculturated with symbols and reasoning skills in a sociocultural setting (Hutto and Myin, 2013, 2017; Durt et al., 2017; Hutto et al., 2020; Satne, 2021). In short, for enactivists, representing requires enculturation and engagement with thinking and inference about a certain state of affairs in the world, i.e., they theorise.

More sophisticated yet is the scientific work of developing models. Trained and skilled as such, scientists can construct models that aim at explaining the behaviour of a system. While it may be the case that these models involve representational structures, such as symbols, information, or data, it does not follow that the system being investigated under the model possesses the properties of the model. Supposing that it does is a theoretical assumption that involves a metaphor between the thing being investigated and the computational model. While the model results from the best intellectual efforts and training of a fully enculturated agent, the neurocognitive activity is biological activity. Holding the metaphor between scientific computational models and neurons that compute representations, enactivists argue, involves a fallacy of attributing full agent capacities to the neurobiological scales. The rejection of the metaphor does not compromise the usefulness and suitability of simulation modelling and Bayesian inference to the investigation and understanding of the brain. The brain *does not care* about the methods scientists use to pattern its activity.

A definition of cognition should not thereby follow from the assumption of the metaphor: for it should be rejected. An understanding of cognition must result, not from computational models but front-loading computational models. As such, it must result from logical reasoning and thereby implicate the least number of assumptions. It should be a principled description of front-loading the design of experiments and models. Maturana (2002) describes cognition in a form that requires minimal assumptions as it follows from what can be observed in the biological system (as opposed to assigning machine-like properties):

‘Cognition [is] the capacity that a living system exhibits of operating in dynamic structural congruence with [its] medium’ (p. 26).

Unpacking the concepts, medium refers to the environment, structural congruence to the specifics of its situation, in ways that can be described as coupled or adapted. Maturana (2002) arrives at such a description, he notes, after he had asked himself, ‘[w]hat should happen in the manner of constitution of a system so that I see as a result of its operation a living system?’. Importantly, in answering the question, he tells us, he is not ‘making a hypothesis about how the system was. [he] was proposing that the relation between the internal dynamics of the system and the result of that internal dynamics in the domain in which [he] observed it, would tell [him] what the system was’ (p. 5, emphasis added). For example, in Maturana’s constructivist view of science, *intentionality*, as well as other cognitive functions, can be seen not as an ontological property of the living system but a scientific construct to label the behaviour of an agent directed towards some goal or object, which the scientist can adjudicate as adequate or inadequate. In more detail, the relation unfolds as follows:

If we see a living system behaving according to what we consider adequate behaviour in the circumstances we observe it, we claim that it knows. What we see in such circumstances underlying the adequate behaviour of the living systems is:

- That the living system under our attention shows or exhibits a structural dynamics that flows in congruence with the structural dynamics of the medium in which we see it; and
- That it is through that dynamic structural congruence that the living system conserves its living (Maturana, 2002, p. 26, emphasis added).

The description of cognition by Maturana (2002) can be formally put as follows:

P1: If we see adequate cognitive behaviour, the system is cognitive.
P2: Adequacy means that a system exhibits dynamics that are congruent with the dynamics of the medium.
P3: The system maintains itself by being congruent with the medium.
Conclusion: The system is cognitive.

This understanding allows then to say something about whether or not a system can be deemed as cognitive, refraining from forcing the definition with suppositions and assumptions following from metaphorical interpretations.

In the same vein, Maturana and Varela (1980) tells us, ‘what [we] call normative activities are not aspects of […] autopoiesis [but only] *commentaries* or *explanatory propositions that [we] make about what [we] may think* that should occur in the […] organism’ (Maturana, 2011, pp. 149–150, emphasis added). Notably, enactivists such as Maturana and Varela (1980) stress that scientific modelling and explanations belong to the scientific realm. To put it more precisely, when we use a computational model to make sense of behaviour, both the properties constructed and the interpretations made by the use of the model are not aspects of the system of scientific scrutiny. This is precisely why this specific metaphor – between the brain and a computer – is misleading: ‘representation’ is not to be found in the brain but as a representation of the scientist herself constructing, testing, and interpreting the results of an experiment theory.

While the notion of representation is widely used in cognitivist neuroscience, it is not endorsed across the board in neuroscience. Skarda (2013, p. 265) notes that ‘recent findings in neurophysiology and cognitive science point to the conclusion that cognition can be explained without appeal to the representations and rules of earlier cognitivist explanations’. This claim is motivated by 30 years of research together with Freeman (2001). One example of this research is their experiments on odour, that the idiosyncrasy of neural activity cannot be best described as a computation of a domain-specific system that automatically activates according to a programme of certain structural rules (a machine-like system). They remark that ‘there is nothing intrinsically representational about this dynamic process until the observer intrudes. It is the *experimenter who infers what the observed activity patterns represent* to or in a subject, to explain his results to himself’ (Freeman and Skarda, 1990, p. 4, emphasis added).

As opposed to the interpretations made by the experimenter, properties of the system should be laid out carefully not to involve experimental theories. One feature that is referred to throughout and undeniable in neurocognitive activity is that it is dynamic and lives in high dimensionality, i.e., it is a complex system. There is good reason, then, to think that, to know what something is, it is insightful to investigate its behavioural dynamics, conceptually and mathematically. Here is where philosophy of mind and modelling science can fruitfully meet.

Freeman (2001) demonstrates a mechanism for embodied cognition ‘casting the mental events involved in perception, planning, deciding, and remembering in the same analogic dynamical language as used to describe bodily movement’. Applying the vocabulary of movement to perceiving, planning, deciding, or remembering as moving around or navigating an environment (as opposed to internal information-based processes and representations) remarkably breaks up with cognitivism and, together with his findings for neural activity as self-organising dynamics (Kozma and Freeman, 2017), is perfectly aligned with the most recent research in EECS and Dynamical Systems Theory (DST).

Dynamical Systems Theory is well-equipped with the computational tools that allow simulating neurocognitive activity as a dynamical system. The basis is the insight that where ‘rather than computation, cognitive processes may be dynamical systems; rather than computation, cognitive processes may be state-space evolution within these very different kinds of systems’ (Van Gelder, 1995, p. 346). Neurocognitive activity ‘lives’ in high dimensions. More precisely, its dynamics occur in dimensions with high degrees of freedom for having too many attributes in parameter spaces or configuration spaces. High-dimensional spaces arise within the context of modelling data sets with many attributes.

The following section offers a generative model under DCM that explains neural activity as the covariance between the data obtained and how the data was generated given its dynamical and complex nature.

## Dynamical Causal Model of Neurocognitive Activity

This last section focuses on a model that links neuroimaging data and the theory (parameters) under the procedure explained in section Scientific Models in Neuroimaging, i.e., Dynamical Causal Modelling (DCM). As we will see, under DCM, neurocognitive activity is explained, not as machine-like computations, but dynamic interactions of flows and influences underlying neurobiological signalling.^9^

Generally speaking, a generative model is a model of the conditional probability of data given parameters, P(V|A) = y [as opposed to an inference model of the conditional probability of the data given the observations, P(A|V) = x]. The generative model is solving for model evidence, where the probability of the model is the probability of data given parameters multiplied by the probability of the parameters, where P(A) is the model evidence.


P(V,A)=P(V|A)P(A)

Zarghami and Friston (2020) recently offered a dynamic model^10^ for effective connectivity.^11^ Extending an established spectral DCM (Friston et al., 2003, 2019; Daunizeau et al., 2011; Razi and Friston, 2016), to generate neuronal functional connectivity data features that change over time. The goal is to map these (observable and) evolving spatiotemporal patterns back to the (unobservable) evolving neuronal networks that generate them. This can be achieved by constructing generative models of the observations and adopting an inference procedure to estimate the time-varying effective connectivity of neuronal activity.

The brain continuously expresses transient patterns of coordinated activity that emerge and dissolve in response to internal and external perturbations. The emergence and evolution of such metastable coordination dynamics (in self-organising complex systems, such as the brain) is nonlinear. Given this, the scientific goal is to identify the model (network) with the greatest evidence for any given data and infer the corresponding (context-sensitive) coupling or effective connectivity parameters.

**Figure 2:**
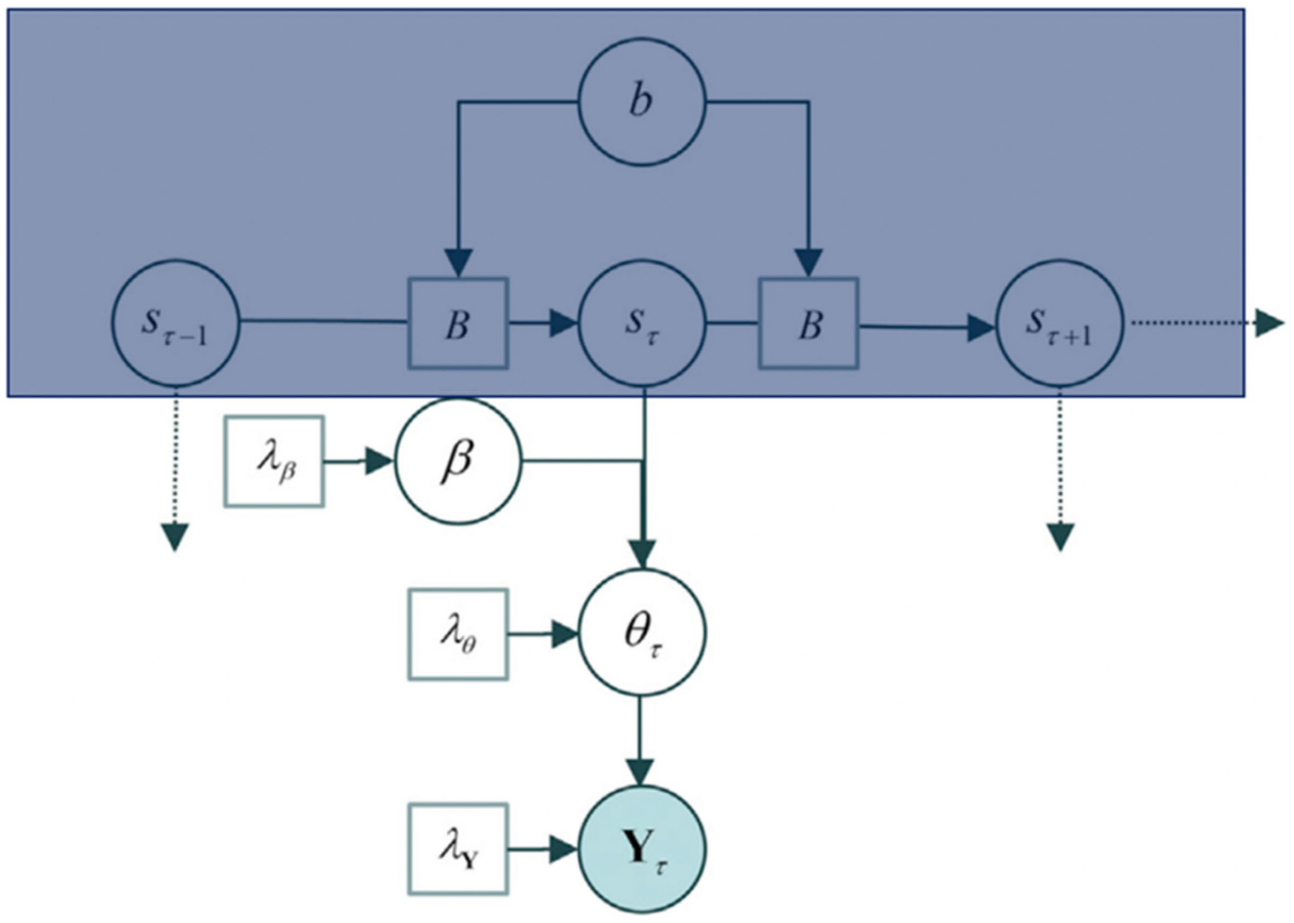
The generative model P (Y, η), where *η* = (b, s, β, θ, and λ). *b* refers to the parameters for the matrix B, where the Matrix B comprehends the posterior expectations, s refers to the hidden states, *β* to the connectivity parameters, θ to the connectivity patterns, and Y to the cross-spectral data features of an epoch that is, the cross-correlation/variation between two time series (s, θ). The model is built up to explain the data, Y. The box in blue corresponds to a hidden Markov blanket (further detail in Figure 3). Figure modified from Zarghami and Friston (2020).

The generative model underlying itinerant brain states is based on the contention that macroscopic (slow) dynamical modes (Haken, 1983; Jirsa et al., 1994) visit a succession of unstable fixed points in the parameter space of directed connectivity. This model departs from the hypothesis that neuronal dynamics are generated by patterns of intrinsic (within a region) and extrinsic (between regions) connectivity changes over time. It does so by assuming that a heterocyclic orbit can trace the patterns of neuronal connectivity^12^ and that the transitions from one stable point to the other are fast (more detail below). This allows then to attain the main component for developing a model under variational Bayes: parameters. Figure 2 presents the generative model of effective connectivity with such parameters.

The goal of setting up the generative model is to solve for Y. The generative model proceeds as follows: (1) Sampling of a state transition matrix from a distribution parameterised by its concentration parameters b. (2) The transition matrix B is then used to select the current brain state given the previous state, assuming a small number of hidden states (s). (3) The current state then selects a state-specific connectivity pattern according to β sampled from a multivariate Gaussian distribution. (4) Further, a random Gaussian variate is added to the pattern to generate connectivity (θ) for the current epoch. This connectivity allows moving forward to define the neuronal network (transfer functions and cross spectra) under local linearity assumptions and parameterised, scale-free, endogenous fluctuations in each brain region, Y.

**Figure 3:**
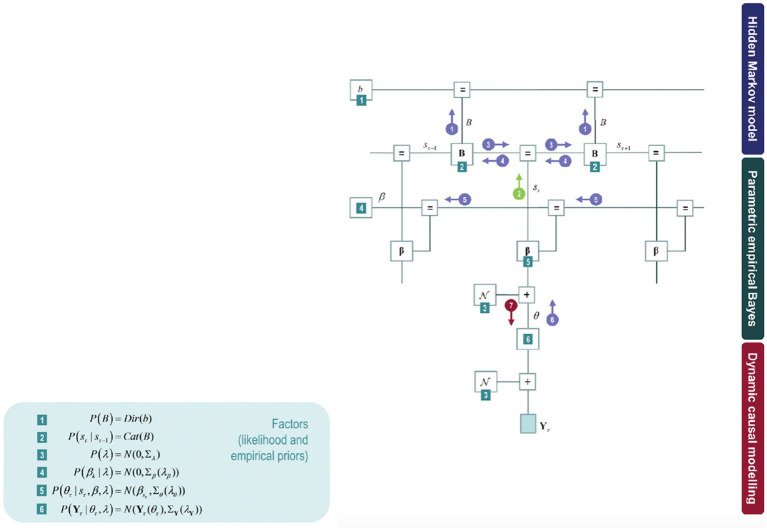
The upper part of the model corresponds to a hidden Markov blanket (highlighted in Figure 2 in a blue box) on the left panel. The middle part of the model fits to the empirical priors (parametric empirical Bayesian scheme). The lower part of the model refers to a conventional DCM analysis of complex cross spectra within each epoch. The DCM parameter estimates then constitute the evidence for a hierarchical model of changing connectivity over epochs, estimated using parametric empirical Bayes (PEB). Some factors can be found on the right panel throughout the model (green numbered squares), corresponding to the parametric empirical Bayes.

This model can further be expanded by model inversion with variational message passing that emerges from free energy minimisation: identifying the relations among the model components *as if* each component were sending a message to the next. Figure 3 shows these messages in circles with arrows amongst the components of the model: the posterior expectation of the transition matrix (B), the posterior expectation of hidden states (s), posterior state-specific connectivity patterns (β), and posterior epoch-specific connectivity (θ).

Constructing a variational free energy bound on model evidence (also known as an ELBO in Machine Learning) makes it possible to attain a functional of the observed data and an approximate posterior density. The data corresponds to the cross spectra (Y) constituting an fMRI time series. The approximate posterior is obtained by a mean-field approximation over all unknown parameters (β, b) and states (s), denoted by Q. The approximate posterior is then optimised for the free energy functional, i.e., by minimising the KL divergence between the approximate and the real or true posterior (mirroring the steps explained in the previous section).

This optimisation allows to further obtain a lower bound on model evidence for the free energy. This means that we can use the free energy functional for model comparison regarding evidence or marginal likelihood. It is possible to compare different models with different hidden Markov blankets (depicted in Figure 3 with a blue box). In conclusion, optimising variational free energy can be used for Bayesian model selection.

An initial assumption is to suppose the hidden Markov model is motivated by orbits. That is to say, that the connectivity parameter space will visit a succession of unstable fixed points (Rabinovich et al., 2008, 2012). This allows us to draw two further assumptions. It assumes that connectivity patterns trace out a heterocyclic orbit – a stable heteroclinic cycle (SHC) in the parameter space visiting a discrete number of unstable fixed points in a winless competition among states of brain activity (Afraimovich et al., 2011; Deco and Jirsa, 2012). An SHC is a set in the phase space of a dynamic system consisting of a circle of equilibrium points and connecting heterocyclic connections. If a heterocyclic cycle is asymptomatically stable, approaching trajectories spend longer periods in a neighbourhood of successful equilibria. However, in high dimension connections, approaching trajectories will not persist due to a large number of parameters. The second assumption is that the transitions from one unstable fixed point to the next are fast regarding the time connectivity remains in the neighbourhood of a fixed point. This assumption leverages from features of the SHC: (i) the origin of the structural stability and (ii) the long passage time in the vicinity of saddles^13^ in the presence of moderate noise in high dimension connections with large degrees of freedom (Rabinovich and Varona, 2018). These assumptions further allow for inferring attributes that the probability transition matrices must possess: (1) *dwell prior*: the dwell time at an unstable fixed point must be short; (2) *orbit prior*: there will be a systematic structure with predictable transitions from one unstable fixed point to the next; and finally (3) *equidistribution prior*: the hidden states required to explain the prior must be all occupied in a non-trivial way in time over which the data is observed.

In conclusion, a generative model is constructed to explain Y. Y is the cross spectra or covariance between a time series of states and an fMRI time series of connectivity. A mean-field approximation obtains the approximate posterior over all unknown parameters (β, b) and states (s). The approximate posterior is then optimised concerning the free energy functional, i.e., by minimising the KL divergence between the approximate posterior and the real or true posterior with a variational free energy bound on model evidence (between competing models with different Markov blankets). To overcome the posterior parameter space’s ill-conditioned information geometry, an assumption is introduced: the hidden Markov blanket is motivated by orbits. From this, a set of attributes is inferred about the transition matrices, which allows for inferring how the data observed in the fMRI times series was generated. Crucially, this means that, although the model does not represent the target – the model ‘lives’ in low dimension as opposed to the activity aimed to be explained, ‘living’ in high dimension – it has predictive and explanatory power. More precisely, it allows for drawing explanations as well as predictions about neuronal activity. Lastly, using DCM delivers a profile and understanding of neurocognitive activity as enactive and dynamically situated.

## Conclusion

This paper rejected the analogy between neurocognitive activity and a computer. It was shown that the analogy results from assuming that the properties of the models used in computational cognitive neuroscience (e.g., information, representation, etc.) must also exist in the system being modelled (e.g., the brain). In section Scientific Models in Neuroimaging, we have seen how computational models offer a link between the collected data (e.g., behavioural or neuroimaging) and a possible explanation of how it was generated (i.e., a theory). While the usefulness of computational models is unquestionable, it does not follow that neurocognitive activity should literally possess the properties used in the model (e.g., information, representation). The last section offered an alternative account of neurocognitive activity bringing together the EECS and the formalisms of DCST. While both these accounts individually reject the mind as a computer metaphor, they rarely explicitly work together. The last section has shown how the cooperation between EECS’s characterisation of cognition and DCST’s formalisms offers a sustained and cohesive programme for explaining neurocognitive activity, not as a machine but as a biologically situated organism. Mainly this link was made by focusing on DCM of neurocognitive activity.

## Data Availability Statement

The original contributions presented in the study are included in the article/supplementary material, further inquiries can be directed to the corresponding author.

## Author Contributions

The author confirms being the sole contributor of this work and has approved it for publication.

## Conflict of Interest

The author declares that the research was conducted in the absence of any commercial or financial relationships that could be construed as a potential conflict of interest.

## Publisher’s Note

All claims expressed in this article are solely those of the authors and do not necessarily represent those of their affiliated organizations, or those of the publisher, the editors and the reviewers. Any product that may be evaluated in this article, or claim that may be made by its manufacturer, is not guaranteed or endorsed by the publisher.

## Footnotes

^1^Cognitivism is the programme that states that cognition is information processing, i.e., rule-based manipulation of symbols. It works through any device which can support and manipulate discrete physical elements: the symbols. A system is functioning adequately as cognitive when the symbols appropriately represent some aspect of the real world, and the information processing leads to a successful solution of the problem posed to the system (Varela, 1992, p. 239).

^2^This has been due mainly to the discovery and introduction of Diffusion Tensor Imaging (DTI), which serves as a non-invasive tool that allows magnetic scanning of the orientation of water molecules. It becomes possible, for the first time, to map neural structures in remarkable detail. DTI is helpful to investigate the connectivity in cognitive networks (Le Bihan et al., 2001; Skudlarski et al., 2008; Wang et al., 2020).

^3^See the distinction between effective and functional connectivity in Friston (2011).

^4^In dynamic causal modelling – based upon dynamical systems theory – causality has a technical meaning. In this setting, the generative models or hypotheses about dynamical architectures are causal in a control-theoretic sense. This means that all models or hypotheses are stipulative about causal (dynamic) architectures.

^5^However, note that the idea of firing neurons in the nervous system could be modelled as logical propositions date back to McCulloch and Pitts (1943).

^6^See how Egan (2020) challenges teleosemantics.

^7^See also McCulloch and Pitts (1943); Smolensky (1988); Rumelhart (1989).

^8^Clark (2015) holds a similar perspective: at every level of a Bayesian hierarchy, he supposes a two-step predictive processing involving an initial issue of a prediction (representation) that is then updated by a prediction error (representation). See Orlandi and Lee (2019).

^9^Some might call this a model-like biological computation, which would be acceptable, as long as understood, not as a machine-like computation.

^10^Refer to the glossary for concepts typically employed in dynamical systems theory.

^11^For a review on effective connectivity, see Friston (2011).

^12^A heteroclinic orbit is a path in phase space that joins two different equilibrium points.

^13^A saddle point or minimax point is a point on the surface of the graph of a function, where the slopes (derivatives) in orthogonal directions are all zero (a critical point) but which is not a local extremum of the function.
